# SARS-COV-2 Infection, Vaccination, and Immune-Mediated Diseases: Results of a Single-Center Retrospective Study

**DOI:** 10.3389/fimmu.2022.859550

**Published:** 2022-03-21

**Authors:** Michele Maria Luchetti Gentiloni, Valentino Paci, Valentina Marconi, Marco Gigli, Devis Benfaremo, Raffaella Sordillo, Cristina Macchini, Leonardo Massaccesi, Gian Piero Perna, Anna Maria Offidani, Gianluca Moroncini

**Affiliations:** ^1^ Clinica Medica, Ospedali Riuniti Ancona University Hospital, Ancona, Italy; ^2^ Department of Clinical and Molecular Sciences, Marche Polytechnic University, Ancona, Italy; ^3^ Internal Medicine Residency Program, Marche Polytechnic University, Ancona, Italy; ^4^ Cardiologia Subintensiva, Ospedali Riuniti Ancona University Hospital, Ancona, Italy; ^5^ Clinica Dermatologica, Ospedali Riuniti Ancona University Hospital, Ancona, Italy

**Keywords:** SARS-CoV2, COVID-19, Immuno-Mediated Reactions, autoimmunity, vaccine

## Abstract

**Objectives:**

The relationship between infections or vaccine antigens and exacerbations or new onset of immune-mediated diseases (IMDs) has long been known. In this observational study, conducted during the COVID-19 pandemic, we evaluated the onset of clinical and laboratory immune manifestations related to COVID-19 or SARS-CoV-2 vaccination.

**Methods:**

Four groups of patients were evaluated: A) 584 COVID-19 inpatients hospitalized from March 2020 to June 2020 and from November 2020 to May 2021; B) 135 outpatients with previous SARS-CoV-2 infection, assessed within 6 months of recovery; C) outpatients with IMDs in remission and flared after SARS-COV-2 infection; D) outpatients with symptoms of probable immune-mediated origin after SARS-CoV-2 vaccination.

**Results:**

In cohort A we observed n. 28 (4.8%) arthralgia/myalgia, n. 2 (0.3%) arthritis, n. 3 (0.5%) pericarditis, n. 1 (0.2%) myocarditis, n. 11 (1.9%) thrombocytopenia or pancytopenia, and in the follow up cohort B we identified 9 (6.7%) cases of newly diagnosed IMDs after the recovery from COVID-19. In all cases, serological alterations were not observed.

In cohort C we observed n.5 flares of pre-existing IMD after SARS-COV2 infection, and in the cohort D n. 13 IMD temporally close with SARS-CoV-2 vaccination in 8 healthy subjects (with clinical classifiable IMD-like presentation) and in 5 patients affected by an anamnestic IMD. Also in these latter cases, except in 2 healthy subjects, there were not found serological alterations specific of a classifiable IMD.

**Conclusions:**

This study suggests that the interplay between SARS-CoV-2 and the host may induce complex immune-mediated reactions, probably induced by the anti-spike antibodies, in healthy people and IMD patients without specific serological autoimmunity. Moreover, our data suggest that the anti-SARS-CoV-2 antibodies generated by the vaccination may cause in healthy subjects’ clinical manifestations similar to well-definite IMDs. These findings support the hypothesis that SARS-Cov2 infection in COVID-19 induce an innate and adaptive immune response that may be both responsible of the symptoms correlated with the occurrence of the IMDs described in our study. And, in this context, the IMDs observed in healthy people in close temporal correlation with the vaccination suggest that the anti-Spike antibodies may play a key role in the induction of an abnormal and deregulated immune response.

## Introduction

Relationships between viral infections, vaccine antigens and new onset or exacerbation of Immune-Mediated Diseases (IMDs) have been studied and acknowledged from long time.

Usually, this phenomenon has been attributed to cross-reactivity, where the neutralizing antibodies produced secondary to an antigenic stimulus (viral or vaccine-induced) react towards the body’s self-tissues. Systemic Lupus Erythematosus, Rheumatoid Arthritis, Autoimmune Thrombocytopenia, Multiple Sclerosis, Guillain-Barré Syndrome, and other demyelinating neuropathies can be listed among the most important IMDs associated with autoimmune cross-reactivity mechanisms (1–3).

The pathogen SARS-CoV-2 is no exempt from this mechanism. Since the beginning of the COVID-19 pandemic, SARS-CoV-2 ability to induce auto-antibodies production and IMDs clinical manifestations has been observed in multiple studies, both *in vitro* and *in vivo* (4). Consequences secondary to severe SARS-CoV-2 infection are partly attributable to immune-mediated mechanisms of organ damage too (5, 6).

Furthermore, new and previously unknown autoimmune diseases have been described as a complication of SARS-CoV-2 infection, like the Multisystem Inflammatory Syndrome in Children (MIS-C) (7).

The neutralizing antibodies produced by the human body against SARS-CoV-2 are for the vast majority directed against the Spike protein, responsible for the interaction between the virus and ACE-2 receptor on human respiratory cells. Specifically, the site responsible for binding to the ACE-2 receptor is the Receptor Binding Domain (RBD) region of the Spike protein. Consequently, this region is the target of over 90% of neutralizing antibodies. Vaccines are also based on this mechanism, taking advantage of the production of antibodies against the RBD region (8).

Considering the above, the possibility that anti-Spike antibodies could potentially cross-react and cause autoimmune reactions is at least intriguing.

Besides, patients with IMDs, or with familial or genetic predisposition to autoimmunity, have shown greater susceptibility than the general population to manifest COVID-19 related autoimmune reactions (9).

Furthermore, currently few studies adequately investigated the correlation between vaccination and the development of IMDs or the induction of a flare of a pre-existing disease (10–12). Considering the possible role of anti-Spike antibodies in autoimmune manifestations related to SARS-CoV-2 infection, it is worth hypothesizing that even the vaccine-induced antibodies may seldom cross-react, triggering immunological manifestations (13).

If this hypothesis is confirmed, it should lead to a more careful evaluation of the risks and benefits of vaccination in young patients with a predisposition to develop autoimmune reactions.

## Materials and Methods

### Patients

Four groups of patients were evaluated in an observational retrospective analysis as follows:

A) patients affected by COVID-19, and B) patients recovered from COVID-19, in outpatient follow up; C) patients affected by flare of IMDs after COVID-19; and D) flare of IMDs or onset of IMD after vaccination anti-SARS-CoV-2.

The primary objective of this single-center retrospective study, conducted during the COVID-19 pandemic, is to assess the onset of clinical and laboratory immune manifestations related to SARS-CoV-2 infection or vaccination.

The primary endpoint of the study was the occurrence or flare of IMDs:

a) in patients affected by acute COVID-19;

b) in patients recovered and in outpatient follow up after COVID-19;

c) in patients affected by anamnestic IMD after infection by SARS-Cov2;

d) in close temporal correlation with anti-SARS-Cov2 vaccination in patients affected by an anamnestic IMD or healthy subjects.

Secondary endpoints of the study were:

▪ clinical characterization,

▪ laboratory findings, and

▪ clinical outcomes after the therapy,

of the IMDs occurring in the groups listed above.

**Figure 1:**
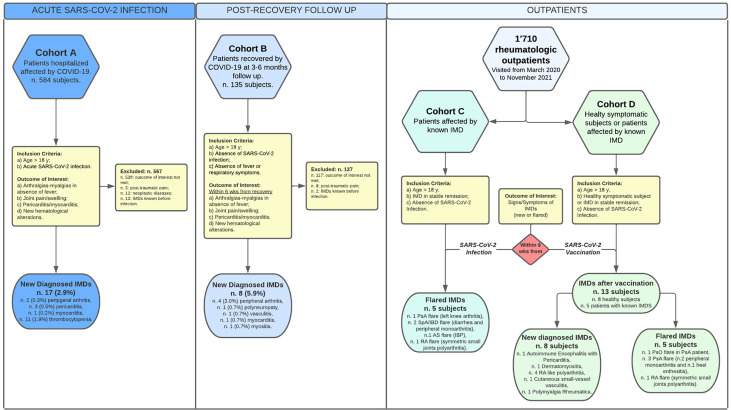
Schematic representation of the study and patients’ groups. In the flow chart are represented the composition of the patients’ cohorts/groups, inclusion/exclusion criteria in the study and brief presentations of the clinical outcomes. All the patients who suffered of IMDs symptoms after the vaccination had been vaccinated with two boost doses of the vaccines listed in the **Table 6**. For more details, see the *Materials and Methods* section.

A schematic representation of the study and the patients’ clinical characteristics are shown in **Figure 1** and **Table 2**, respectively.

**Table 1:** Descriptive characteristics of the patients hospitalized for COVID-19.

Variables	Cohort A (n. 584)	Cohort B (n. 135)	p value
**Age (yrs ± SD)**	62.42±15.78	61.71±15.57	n.s.
**Sex (M/F, %)**	62/38	69/31	n.s.
**Smokers, n (%)**	372/584 (56)	69/135 (51)	n.s.
**COVID-19 clinical, moderate, n (%)^1^**	490/584 (83.9)	64/135 (48)	n.s.
**COVID-19 clinical, severe, n (%)**	82/584 (14.04)	21/135 (15.55)	n.s
**COVID-19 clinical, critical, n (%)**	12/584 (2.06)	3/135 (2.22)	n.s.
**Hypertension, n (%)**	362/584 (62)	69/135 (51)	n.s.
**Diabetes, type II, n (%)**	280/584 (48)	55/135 (41)	n.s.
**COPD, n (%)**	239/584 (41)	51/135 (38)	n.s.
**Cardiovascular Disease^2^, n (%)**	204/584 (35)	55/135 (41)	n.s.
**Renal Failure, n (%)**	198/584 (34)	39/135 (29)	n.s.
**Dyslipidemia, n (%)**	415/584 (71)	92/135 (68)	n.s.
**Connective disease^3^, n (%)**	13/584 (2.3)	2/135 (1.5)	n.s.
**Neoplastic disease^4^, n (%)**	12/584 (2.05)	0/135 (0)	n.s.
**Psychiatric disorder^5^, n (%)**	26/584 (4.5)	4/135 (3)	n.s.
**Neurologic disease^6^, n (%)**	38/584 (6.5)	9/135 (6.6)	n.s.

^1^The clinical spectrum of COVID-19 correspond to the definition of the National Institute of Health of the United States of America (NIH) COVID-19 treatment guidelines (14). ^2^Ischemic chronic heart disease, chronic cardiac failure, hypertensive cardiopathy. ^3^Patients affected by anamnestic connective disease (n.12 rheumatoid arthritis, n. 1 scleroderma, n. 1 undifferentiated connective disease). ^4^Patients affected by anamnestic neoplastic disease (n. 5 lung neoplasia, n. 5 breast carcinoma, n. 2 sarcoma). ^5^Patients affected by anamnestic psychiatric disease (n.12 severe depression, n. 14 psychotic disease, n. 4 bipolar disease). ^6^Patients affected by anamnestic neurologic disease (n. 35 dementia, n. 12 Alzheimers’ disease). Statistic: Categorical data were summarized using absolute frequencies and percentages, while continuous data were summarized using mean ± SD. Comparisons between groups were assessed by means of t test for independent samples or Chi square test, as appropriate. A p < 0.05 was considered significant; if 0.05: p not significant (n.s.). Yrs, years; M/F, males/females; COVID-19, COronaVIrus Disease 2019; COPD, chronic obstructive pulmonary disease.

**Table 2:** Immune-Mediated Diseases (IMDs) diagnosed in patients hospitalized for COVID-19.

Parameter	Value [n/tot. number(%)]
Sex	
Female (52,9%)	9/584 (1.54%)
Male (47,1%)	8/584 (1.37%)
Age (mean)	60,23
IMDs	
Pericarditis	3/584 (0.5%)
Myocarditis	1/584 (0.2%)
Arthritis	2/584 (0.3%)
Thrombocytopenia or Pancytopenia	11/584 (1.9%)

In the cohort A we enrolled 584 patients with moderate to severe COVID-19 disease, hospitalized in the “COV-4” ward of the hospital “Ospedali Riuniti di Ancona” (AOU), Ancona, Italy, from March 2020 to June 2020 and from November 2020 to May 2021 (**Table 1**).

Inclusion criteria were the following: *a)* age>18 years (yrs); *b)* acute infection by SARS-Cov2 detected with at least two nasopharyngeal swabs and by real-time reverse transcription polymerase chain reaction method.

Exclusion criteria were the following: absence of the inclusion criteria; trauma; neoplastic diseases; non-organ specific connective tissue diseases.

The clinical spectrum of the patients hospitalized for COVID-19 was achieved following the National Institute of Health of the United States of America (NIH) COVID-19 treatment guidelines as follows: a) Moderate Illness: Individuals who show evidence of lower respiratory disease during clinical assessment or imaging and who have an oxygen saturation (SpO2) ≥94% on room air at sea level; b) Severe Illness: Individuals who have SpO2 <94% on room air at sea level, a ratio of arterial partial pressure of oxygen to fraction of inspired oxygen (PaO2/FiO2) <300 mm Hg, a respiratory rate >30 breaths/min, or lung infiltrates >50%; c) Critical Illness: Individuals who have respiratory failure, septic shock, and/or multiple organ dysfunction (14).

All the patients were assessed with clinical examinations and laboratory test daily and with High-resolution computed tomography (HRCT) in the emergency department and during the hospitalization, depending on the clinical evolution.

In the cohort B we evaluated 135 outpatients from cohort A discharged out of the hospital and evaluated within 6 months of the recovery by COVID-19.

Inclusion criteria in the cohort B were the following: *a)* age>18 yrs; *b)* absence of SARS-Cov2 detected with at least two nasopharyngeal swabs and by real-time reverse transcription polymerase chain reaction method; c) absence of any respiratory symptom and fever for at least one week.

The follow up examinations were scheduled at 3 and 6 months from the discharge and included clinical examinations, laboratory test and HRCT. The scheduled follow up was anticipated in case of the occurrence of any clinical symptom.

The outcome of interest in cohort A and B was the onset of: a) symptoms of rheumatic diseases (arthralgias-myalgias) in absence of fever; b) articular pain and/or joint swelling; c) chest pain and pericarditis/myocarditis confirmed by laboratory test (elevation of erythrocyte sedimentation rate, ESR; C-reactive protein, CRP; creatin-kinase, CK; high-sensitivity troponin, hs-Tpn, electrocardiographic recording, and ultrasound-doppler echocardiography; d) clinical signs of involvement of the central and/or peripheral nervous system; e) hematological alterations, not reported in the clinical history.

The cohorts C and D were constituted by two group of patients evaluated for IMDs symptoms among the whole cohort of 1710 outpatients evaluated in our rheumatologic clinic for rheumatologic symptoms from March 2020 to November 2021.

The cohort C was constituted by patients affected by IMD (among the 849 IMD patients in our center currently in follow up for at least 2 years) and satisfying the following inclusion in criteria: *a)* age >18 yrs; b) IMD in stable remission for at least 6 months.

The cohort D was constituted by subjects reporting IMD symptoms or patients affected by flare of IMDs in temporal close correlation with vaccine administration.

The inclusion criteria in cohort D were the following: *a)* age>18 yrs; b) IMD in stable remission for at least 6 months OR healthy subjects without IMD symptoms before the vaccination; *c)* absence SARS-Cov2 detected with at least two nasopharyngeal swabs and by real-time reverse transcription polymerase chain reaction method; d) SARS-COV2 vaccination, less than 6 weeks before the onset of the following symptoms: arthralgias-myalgias; articular pain and/or joint swelling; chest pain; dermal lesions or signs of skin inflammation; clinical signs of involvement of the central and/or peripheral nervous system.

The outcome of interest in cohorts C and D was occurrence of IMD flare or the development of new signs/symptoms of IMD. Data were collected by consulting inpatient and outpatient medical records.

The diagnosis of SARS-Cov2 acute infection in all the patients included in the study was made with at least two nasopharyngeal swabs and by real-time reverse transcription polymerase chain reaction method.

The diagnosis of IMDs was carried out by an experienced team of physicians through history, physical examination, and laboratory tests (see the following section).

Immune-Mediated Diseases (IMDs) were considered all the diseases that involve an immune response that is inappropriate or excessive, and is caused, signified, or accompanied by dysregulation of the immune system, with/without the presence of disease-specific autoantibodies (15, 16).

Given that the most common post-infectious or post-vaccinal rheumatological IMD is Reactive Arthritis, and considering that, according to the American College of Rheumatology Diagnostic Criteria (1999), it must occur within 6 weeks of the infectious trigger, we considered relevant only immunological manifestations occurred within 6 weeks from infection or vaccination.

### Laboratory Tests

Apart from clinical manifestations, we also collected data regarding routine laboratory tests including complete blood count, alanine transferase (ALT), aspartate transferase (AST), creatinine, uric acid, erythrocyte sedimentation rate (ESR) and C-reactive protein (CRP), as available.

If available, we also recorded results of anti-nuclear antibodies (ANAs), anti-cytoplasmic ANCA (c-ANCA) and anti-perinuclear ANCA (p-ANCA) antibodies, rheumatoid factor and anti-citrullinated peptides antibodies (ACPAs), and anti-Ro, anti-La, anti-double strand DNA and anti-Sm autoantibodies.

Any other relevant laboratory or instrumental investigation that had been required for the diagnosis was also recorded.

### Ethical Approval and Data Analysis

The study was carried out in compliance with the Declaration of Helsinki on ethical principles for medical research. Formal approval by the local Ethics Committee (Comitato Etico Regionale delle Marche) was waived due to the retrospective nature of the study.

All data were transferred to an electronic database and analyzed descriptively using the common analysis methods.

Categorical data were summarized using absolute frequencies and percentages, while continuous data were summarized using mean ± SD. Comparisons between groups were assessed by means of t test for independent samples or Chi square test, as appropriate. A p < 0.05 was considered significant.

## Results

### New Diagnosis of IMDs in Cohort A

New-onset IMDs were observed in a large group of patients affected by COVID-19 and hospitalized in a COVID ward (cohort A).

Among 584 patients non previously diagnosed with IMDs, 17 (2.9%) received a diagnosis of IMD (9 females, 52.9%; 8 males, 47.1%; mean age 60.23) (**Table 2**).

These reactions were further subclassified as n. 2 (0.3%) peripheral arthritis, n. 3 (0.5%) pericarditis, n. 1 (0.2%) myocarditis (severe), n. 11 (1.9%) thrombocytopenia and/or pancytopenia (**Tables 2**, **3**).

**Table 3:** Immune-Mediated Diseases (IMDs) diagnosed in patients hospitalized for COVID-19.

Case	Age	Sex	Clinical Presentation	Laboratory Test^a^	Therapy^b^	Outcome
M.R.	56	M	Arthritis of the I MTF	ESR 45 mmh; CRP 6 mg/dl.	Prednisone	Remission in 2 wks
M.L.B.	86	F	Arthritis of the I MTF	ERS 90 mmh. CRP 6.6 mg/dl.	Prednisone	Remission in 2 wks
I.G.	82	F	Pericarditis	ERS 120 mmh, CRP 33 mg/dl.	Prednisone	Remission in 4 wks
M.O.	67	F	Pericarditis	ERS 99 mmh, CRP 8.8 mg/dl.	Prednisone	Remission in 4 wks
Y.F.	35	F	Pericarditis	ERS 42 mmh. CRP 5.5 mg/dl.	Prednisone	Remission in 4 wks
M.P.	52	F	Myocarditis, severe.	ESR and CRP above normal values	Prednisone; MMF	Remission in 24 wks
M.G.	58	M	Thrombocytopenia	ERS 2 mmh, CRP 1.3 mg/dl	Prednisone	Remission in 2-4 wks in all patients
D.E.	45		Pancytopenia	ERS 2 mmh, CRP 4.5 mg/dl		
P.M.	57		Pancytopenia	ERS 72 mmh, CRP 1 mg/dl		
M.D.	63		Thrombocytopenia	ERS 65 mmh, CRP 18.6 mg/dl		
F.P.	62		Thrombocytopenia	ERS 23 mmh, CRP 15 mg/dl		
I.M.R.	47		Thrombocytopenia	ERS 19 mmh, CRP 2.8 mg/dl		
C.R.	74		Thrombocytopenia severe	ERS 77 mmh, CRP 10 mg/dl		
S.G.	42	F	Pancytopenia	ERS 45 mmh, CRP 8.2 mg/dl	Prednisone	Remission in 2-4 wks in all patients
F.E.K	72		Thrombocytopenia	ERS 16 mmh, CRP 8.1 mg/dl		
M.B.	66		Thrombocytopenia	ERS and CRP above normal values		
L.Z.	60		Thrombocytopenia	ERS 16 mmh, CRP 4.8 mg/dl		

Age: years. Sex: M, male, F, female.

a Laboratory Test: in all the patients the serum levels of anti-nuclear antibodies (ANAs), anti-cytoplasmic ANCA (c-ANCA) and anti-perinuclear ANCA (p-ANCA) antibodies, rheumatoid factor and anti-citrullinated protein antibodies (ACPAs), and anti-Ro, anti-La, anti-dsDNA, anti-Sm autoantibodies were tested and resulted in the normal range.

b Prednisone was administered at the dose of 25 mg/day, scaling the dose until the stop in 2-4 weeks, in all the patients. In case n. 6 prednisone was administered at the dose of 25 mg/day for one week, scaling the dose in 8 weeks to the minimum dose of 4 mg/day and, because the patient was still symptomatic, mycophenolate mofetil was added at the dose of 1 gr twice a day. Wks, weeks; ESR, erythrocyte sedimentation rate; CRP, C-reactive protein; MMF, Mycophenolate mofetil;

Clinical manifestations were mostly acute and observed during hospitalization, with a mean delay of symptom onset of 11 +/- 4 days. Other confounding factors such as joint trauma, additional concomitant infectious triggers or neoplasms were excluded.

Twenty-eight (4.8%) COVID-19 patients of the cohort A reported non-specific musculoskeletal symptoms (myalgia and arthralgia) but did not fulfill criteria for a defined IMD.

These newly reported IMDs were mostly mild to moderate in severity and resolved within 2-4 weeks with restitutio ab integrum after corticosteroids therapy, except for one case of severe myocarditis and one case of severe thrombocytopenia.

In all the patients, ESR and CRP were elevated but the autoantibodies that were tested resulted negative.

### New Diagnosis of IMDs in Cohort B

Cohort B was constituted by outpatient patients of the cohort A in follow-up after the discharge from the COVID ward, evaluated for suspected IMD occurring within 6 weeks from COVID-19.

Eight patients (5.93%) reported symptoms suspect for IMDs. The following diagnoses were made: n. 4 (3.0%) peripheral arthritis, n. 1 (0.7%) polyneuropathy, n. 1 (0.7%) vasculitis, n. 1 (0.7%) myocarditis, n. 1 (0.7%) myositis (**Table 4**).

**Table 4:** Immune-Mediated Diseases (IMDs) diagnosed in outpatients previously hospitalized for COVID-19 and in follow up.

Case	Age	Sex	Timing	Clinical Presentation	Laboratory test^a^	Therapy^b^	Outcome
G.V.	74	M	7	Polineuropaty^c^	ESR 30 mmh. CRP 0,5 mg/dl	Prednisone + Gabapentin	Remission in 6 wks
M.R.D.	49	F	10	Synovitis of left ankle	ESR and CRP above the normal values	Prednisone	Remission in 8 wks
M.B.	52	M	2	Psoriatic arthritis, peripheral^d^	ESR 50 mmh, CRP 0,2 mg/dl	Prednisone + MTX	Remission in 12 wks
C.G.	39	M	38	Urticaria^e^	ESR 70 mmh, CRP 3 mg/dl.	Prednisone	Remission in 16 wks
O.M.C.	54	F	2	Vasculitis^f^	ESR 23 mmh, CRP 1,7 mg/dl	Prednisone	Remission in 12 wks
M.P.	47	F	4	Myocarditis	ESR and CRP above the normal values	Prednisone	Remission in 3 wks
F.M.	82	F	5	Myositis	ESR 39 mmh, CRP 1,0 mg/dl, CK 3539 U/l	Prednisone	Remission in 8 wks
L.R.	56	F	3	Polyarthritis, symmetric, of the small joints	ESR and CRP normal ANA 1/160	Prednisone	Remission in 4 wks

Age: years. Sex: M, male; F, female. Timing: clinical presentation in days.

a Laboratory Test. Serum levels of erythrocyte sedimentation rate (ESR), C-reactive protein (CRP), anti-nuclear antibodies (ANAs), anti-cytoplasmic ANCA (c-ANCA) and anti-perinuclear ANCA (p-ANCA) antibodies, rheumatoid factor and anti-citrullinated protein antibodies (ACPAs), and anti-Ro, anti-La, anti-dsDNA, anti-Sm autoantibodies were tested. There are shown only the test resulted out of the normal value.

b Prednisone was administered at the dose of 25 mg/day, scaling the dose until the stop in the weeks shown in the table in all the patients. In case n. 3, methotrexate (MTX) was added to prednisone at the dose of 10 mg/week and stopped when patient achieved the clinical remission.

c Diagnosed with electromyographic examination (EMG).

d Patient with a familiar history of psoriasis (father), and previous nail disease evaluated as “psoriatic onychopathy”.

e The diagnosis of urticaria vasculitis was made with skin biopsy (vasculitis with infiltration of neutrophils and eosinophils).

f The diagnosis of vasculitis was made with skin biopsy (result: leukocytoclastic vasculitis). Wks, weeks; ESR, erythrocyte sedimentation rate; CRP, C-reactive protein; MTX, methotrexate.

The events were mostly mild-to-moderate and were successfully treated with low-dose corticosteroids, non-steroid anti-inflammatory drugs (NSAID), methotrexate and gabapentin (**Table 4**).

All the cases described occurred with a mean delay of days 8.9 ± 12 from the discharge from the hospital.

### Flare or Onset of New IMDs After SARS-CoV-2 Infection or Vaccination

The flare of an anamnestic IMD or the onset of a new one was evaluated among the whole cohort of 1710 outpatients evaluated in our rheumatologic clinic (n. 849 IMDs in follow up and n. 861 new outpatients evaluated for rheumatologic symptoms).

In the cohort C we evaluated those patients with a pre-existing diagnosis of an immuno-mediated disease and reporting symptoms of the IMDs after SARS-COV-2 infection.

Among this group, 5 patients (all females, mean age 56.8), corresponding to the 0,58% out of the 849 outpatients in follow up in our rheumatologic clinic, reported flares of the IMDs already known but in stable remission of the disease before the SARS-COV2 infection (**Table 5**). The flares presented as follows: acute arthritis of the left knee in n. 1 psoriatic arthritis (PsA); diarrhea and arthritis of the knee or of the right wrist in n. 2 spondyloarthritis associated to inflammatory bowel disease (SpA/IBD), respectively; inflammatory back pain in n. 1 ankylosing spondylitis (AS); and symmetric polyarthritis of the small joints of the hands in n. 1 rheumatoid arthritis (RA). All the patients were successfully treated adding corticosteroid to the ongoing therapy (**Table 5**).

**Table 5:** Immune-Mediated Diseases (IMDs) flared after COVID-19.

Case	Age	Sex	Timing	Clinical Presentation*	Laboratory Test^a^	Therapy^b^	Outcome
L.B.	54	F	3	Flare of PsA	ESR 45 mmh, CRP 5 mg/dl	Prednisone + bDMARD	Remission in 4 wks
R.B.	53	F	4	Flare of SpA/IBD	ESR and CRP above the normal values	Prednisone + bDMARD	Remission in 3 wks
E.G.	29	F	7	Flare of SpA/IBD	ESR 65 mmh, CRP 8 mg/dl	Prednisone + bDMARD	Remission in 3 wks
M.I.	61	F	2	Flare of Ankylosing Spondylitis	ESR and CRP above the normal values	Prednisone + bDMARD	Remission in 6 wks
A.M.T.	83	F	5	Flare of Rheumatoid Arthritis	ESR 85 mmh, CRP 18 mg/dl	Prednisone + bDMARD	Remission in 6 wks

Age: years. Sex: M, male; F, female. Timing: clinical presentation in days. *The clinical flare of each specific disease is reported in the Result section of the text.

a Laboratory Test. Serum levels of erythrocyte sedimentation rate (ESR), C-reactive protein (CRP), anti-nuclear antibodies (ANAs), anti-cytoplasmic ANCA (c-ANCA) and anti-perinuclear ANCA (p-ANCA) antibodies, rheumatoid factor and anti-citrullinated protein antibodies (ACPAs), and anti-Ro, anti-La, anti-dsDNA, anti-Sm autoantibodies were tested. There are shown only the test resulted out of the normal value.

b Prednisone was added to the biologic-disease-modifying anti-rheumatic drug (b-DMARD; adalimumab in cases 1-3, secukinumab in case 4) at the dose of 25 mg/day, scaling the dose until the stop in the weeks shown in the table. Wks, weeks; ESR, erythrocyte sedimentation rate; CRP, C-reactive protein; MMF, Mycophenolate mofetil; PsA, psoriatic arthritis; SpA/IBD, spondyloarthritis associated with inflammatory bowel disease (Crohn disease).

In the cohort D we evaluated n. 13 outpatients with a newly diagnosed or flared IMD in close temporal correlation with SARS-CoV-2 mRNA vaccine administration (**Table 6**). Before the onset of the symptoms, all the patients had received two boost doses of the vaccines types listed in **Table 6**.

**Table 6:** Immune-Mediated Diseases (IMDs) after vaccination anti-SARS-COV-2.

Case	Age	Sex	Timing	Vaccine	Clinical Presentation*	Laboratory Test^a^	Therapy^b^	Outcome
P.F.	71	F	1	Comirnaty	Polyarthritis, RA-like	ESR and CRP above the normal values	Prednisone	Remission in 4 wks
S.F.	72	M	9	Comirnaty	Enthesitis/tenosynovitis of the shoulder; Polyarthritis, RA-like	ESR 84 mmh, CRP 3 mg/dl.	Prednisone	Remission in 8 wks
A.M.	78	F	15	Comirnaty	Capillaritis of the palmar surface	ESR and CRP above the normal values	Prednisone	Remission in 12 wks
S.M.	38	F	1	Comirnaty	Encephalitis, acute^c^ + myocarditis-pericarditis	ESR and CRP above the normal values; ANA 1/320	Prednisone + Colchicine	Remission in 8 wks
R.T.	61	M	1	Comirnaty	Polyarthritis RA-like	ESR 10 mmh, CRP 27,7 mg/dl	Prednisone	Remission 4 wks
L.M. G.	68	M	5	Comirnaty	Polyarthritis RA-like	ESR 73 mmh, CRP 2.7 mg/dl	Prednisone	Remission 3 wks
A.N.	46	F	7	Comirnaty	Dermatomyositis	ESR and CRP normal Anti-SSA Ro and antiMDA5 positive, CK normal, AST 155 U/l, ALT 117 U/l.	Prednisone + MMF	Remission in 5 wks
O.G.	72	M	3	Comirnaty	Polymyalgia Rheumatica	ESR 68 mmh, CRP 1,6 mg/dl	Prednisone	Remission in 6 wks
G.I.	25	F	3	Comirnaty	Flare of Pso in Ax-PsA	N.S.	Prednisone + bDMARD	Remission in 3 months
F.R.	58	M	30	Comirnaty	Flare of Ax-PsA	ESR 15 mmh, CRP 6.8 mg/dl	Prednisone + bDMARD	Remission in 3wks
T.M.	59	F	10	Comirnaty	Flare of PsA	ESR 26 mm/h, CRP 1,1 mg/dl	Prednisone + bDMARD	Active
L.G.	54	F	1	Comirnaty	Flare of PsA	N.S.	Prednisone + bDMARD	Active
A.M.T.	83	F	3	Comirnaty	Flare of RA	ESR 85 mmh, CRP 18 mg/dl	Prednisone + bDMARD	Remission in 6 wks
L.P.	60	F	14	Comirnaty	Flare of RA	ESR and CRP normal	Prednisone + bDMARD	Remission in 4 weeks

Age: years. Sex: M, male; F, female. Timing: clinical presentation in days. *The clinical flare of each specific disease is reported in the Result section of the text. Before the onset of the symptoms, all the patients had received two boost doses of the vaccines types listed in the table.

a Laboratory Test. Serum levels of erythrocyte sedimentation rate (ESR), C-reactive protein (CRP), anti-nuclear antibodies (ANAs), anti-cytoplasmic ANCA (c-ANCA) and anti-perinuclear ANCA (p-ANCA) antibodies, rheumatoid factor and anti-citrullinated protein antibodies (ACPAs), and anti-Ro, anti-La, anti-dsDNA, anti-Sm autoantibodies were tested. There are shown only the test resulted out of the normal value.

b Prednisone was used alone or added to the oncourse biologic-disease-modifying anti-rheumatic drug (b-DMARD) at the dose of 25 mg/day, scaling the dose until the stop in the weeks shown in the table.

c Diagnosed with magnetic resonance imaging. Wks, weeks; ESR, erythrocyte sedimentation rate; CRP, C-reactive protein; MMF, Mycophenolate mofetil; RA, rheumatoid arthritis; Pso, psoriasis; Ax-PsA, spondyloarthritis associated with psoriasis; PsA, psoriatic arthritis.

**Figure 2:**
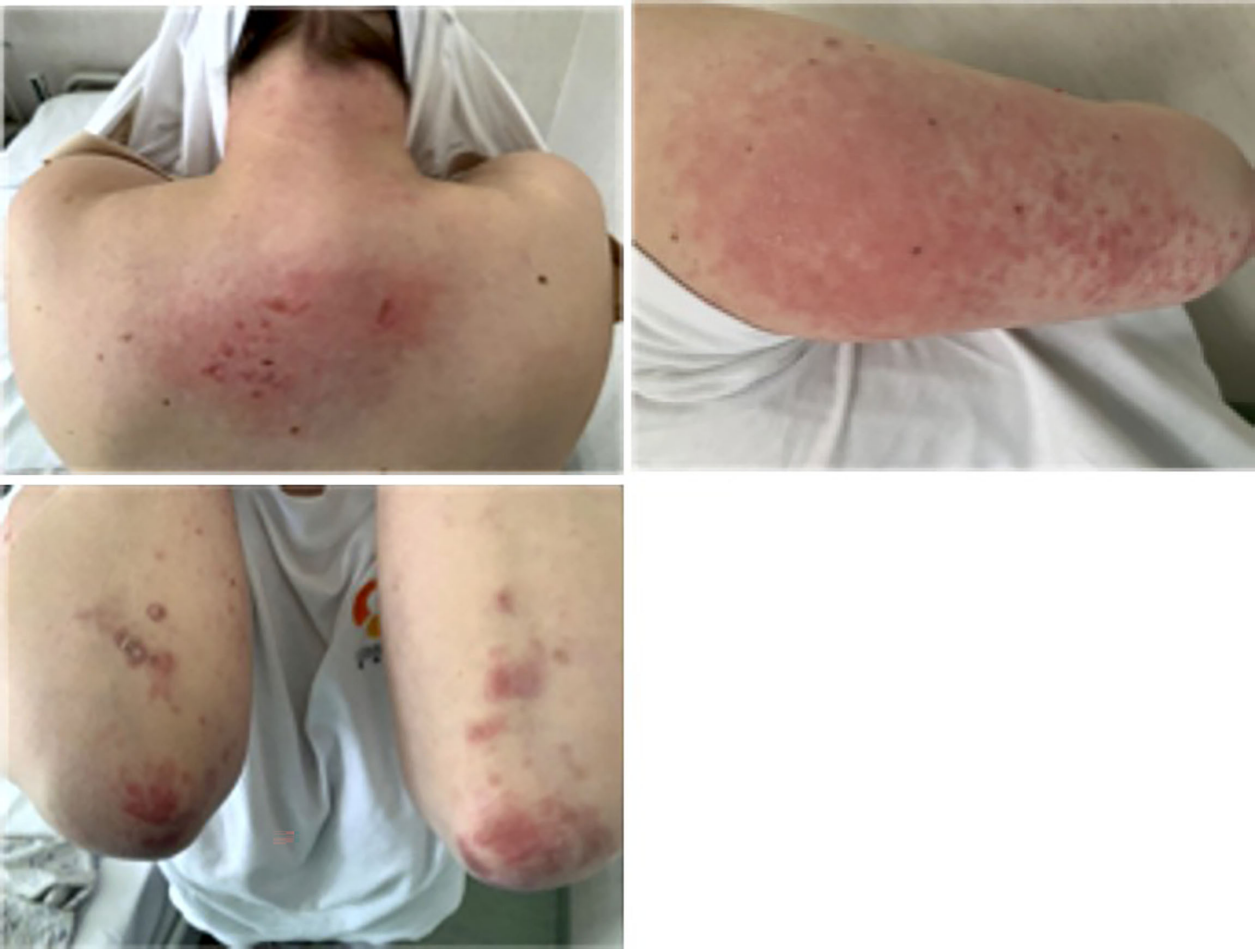
Dermatomyositis-like clinical presentation closely correlated with anti-SARS-2 vaccination. The figure shows a representative case of a dermatomyositis-like clinical presentation in a patient after SARS-COV2 vaccination.

There were observed n. 8 new IMDs, corresponding to the 0.93% among the cohort of 861 outpatients evaluated for new rheumatologic symptoms. These patients showed a heterogeneous clinical spectrum (**Table 6**), including two clinical severe presentations: one patient had autoimmune encephalitis with pericarditis and another one dermatomyositis, with the involvement of the peripheral nervous system (**Figure 2**).

**Figure 3:**
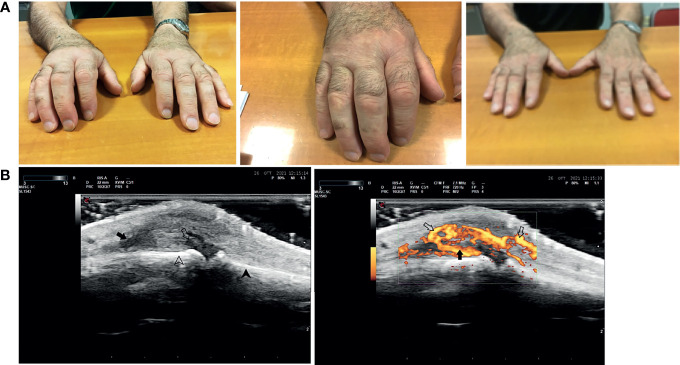
Rheumatoid arthritis-like clinical presentation closely correlated with anti-SARS-2 vaccination. The figure shows a representative case of a rheumatoid arthritis-like clinical presentation in a patient after SARS-COV2 vaccination. Panel **(A)** From left to right: acute phase of the disease, a particular of the right hand, and clinical examination after 4 weeks of corticosteroid therapy. Panel **(B)** Ultrasound doppler examination of the 3rd metacarpal-phalangeal joint of the right hand (from left to right): Grayscale US, Joint capsule (*arrow*) of MCF III dilated by synovial proliferation (*open arrow*) grade III (gs) according to OMERACT criteria. Below are shown the surfaces of metacarpal bone (arrowhead) and proximal phalanx (open arrowhead); Power Doppler Same scan of **Figure 1** with PD showing severly active synovitis (*arrow*) of grade III (pd) according to OMERACT criteria With PD signal becames evident an associated paratenonitis of third digit extensor tendons (open arrows).

In the other patients, n. 4 patients presented a RA-like disease, characterized by symmetric polyarthritis of the wrists and of the small joints of the hands (**Figure 3**), n. 1 had a mild vasculitis of the small vessels of the hands’ fingers, and n. 1 was diagnosed with polymyalgia rheumatica (**Table 6**).

In this cohort, serum autoantibodies were found only in the 2 patients: case n. 4 with autoimmune encephalitis (ANA 1/320) and case n. 6 with dermatomyositis (anti-SSA-Ro-antiMDA5, 1/640).

Vaccination induced a flare of an underlying IMD in 5 patients, corresponding to the 0,58% out of the 849 IMDs in follow up in our clinic, as follows: a flare of psoriasis in one patient with PsA; arthritis of the right knee or of the left wrist in two patients with PsA, respectively; enthesitis of the left Achille’s tendon in one patient with axial-PsA; and symmetric polyarthritis of the small joints of the hands in one patient with RA.

All the patients of the cohort D were successfully treated with corticosteroids, or plus colchicine or mycophenolate mofetil (case n. 4 and n. 6, respectively), or corticosteroids added to the ongoing therapy with a bDMARD in the 2 patients (cases n. 7 and 8) already affected by an IMD (**Table 6**).

## Discussion

Acute coronavirus disease 2019 (COVID-19) pandemic caused by SARS-CoV-2 is characterized by variable clinical presentations, ranging from asymptomatic infection to fatal respiratory failure. Recently, it has become apparent that an exaggerated immune response plays an important role in the pathogenesis of COVID-19, but the intersection of COVID-19 and autoimmunity still needs to be fully elucidated (17).

In fact, it appears that a preexisting autoimmunity may influence, often deleteriously, the course of COVID-19 in certain individuals and, meanwhile, in other patients the virus may contribute to a *de novo* breakdown in immune tolerance, triggering pathogenic immune-mediated clinical manifestations reminiscent of those seen in autoimmune diseases such as antiphospholipid syndrome, inflammatory arthritis, and systemic lupus erythematosus (SLE) (18). In addition, there are numerous case reports of patients developing classifiable autoimmune diseases, such as rheumatoid arthritis, psoriatic arthritis, and type 1 diabetes concomitantly or immediately following SARS-CoV-2 infection (19–22).

As suggested for other viruses (23), the widespread interaction of Coronaviruses with our defense system can trigger autoimmune diseases favored by a molecular similarity between viral and human peptides. Their sporadic transcription and recombination generate a wide number of epitopes that may contribute to elicit autoimmunity trough molecular mimicry, bystander activation, epitope spreading and cytokine storm (24).

In our work, we described a series of cases of IMDs occurring after COVID-19 in a large cohort of patients. We also described a series of cases of well characterized IMDs temporally correlated with anti-SARS-CoV-2 vaccination.

In patients hospitalized for COVID-19, the most observed severe IMDs were diseases of cardiological interest (as pericarditis and myocarditis) and hematological alterations (thrombocytopenia). In the latter case, to improve diagnostic specificity, we considered only cases with platelet counts <100,000/mmc, with a rapid response to steroid therapy and without alternative explanation. According to previous reports (25), thrombocytopenia has been described as an autoimmune manifestation in COVID-19 although without anti-platelet antibodies, likewise in our patients.

In most cases, the hospitalized COVID-19 patients complained mild IMDs symptoms (arthralgia/myalgia) without patent clinical manifestations, whereas a real acute arthritis occurred only in a small percentage of cases.

A subgroup of patients previously affected by COVID-19 and subsequent IMDs were followed-up in the outpatient clinic. Interestingly, these patients reported mainly joint manifestations, more commonly acute exacerbation of seronegative spondyloarthritis (SpA). Acute flares of SpA after COVID-19 have been recently reported by other studies (26).

It is worth noting that all patients described above, although the clinical manifestations of IMDs after COVID-19 closely resembled those of the classifiable disease, did not show the presence of common autoantibodies associated with autoimmune diseases (i.e. SLE or RA), which might have supported the hypothesis of an autoantibody-mediated pathogenesis of the IMDs.

Indeed, we should underline that in these IMDs, putatively correlated with SARS-COV-2 infection, we have not been able to identify, so far, a “disease-tissue”-specific antibody, but it cannot be excluded that in the next future there will be published studies reporting tissue-specific antibodies induced by the SARS-Cov-2 virus.

Moreover, it could be hypothesized that the innate immunity may play an important role in the development of the IMDs in the first phases of the COVID-19 and, subsequently, another important pathogenetic role could be played by the adaptive immunity, namely by the production of the anti-SARS-Cov2 antibodies.

The latter hypothesis, in our study, is supported by the occurrence of the IMDs in close temporal correlation with anti-SARS-Cov2 vaccination and a role of the anti-SARS-COV2 spike antibodies or SARS-COV2-recognizing T cells in triggering a prolonged immune-mediated inflammation in these patients might be postulated.

From this point of view, we additionally reported thirteen cases of new IMDs and five flares of pre-existing IMD that we observed in close temporal correlation with SARS-CoV-2 vaccination with a mRNA vaccine.

The possibility that vaccinations could induce a flare of an underlying rheumatic disease is still controversial and vaccines are generally considered to be safe (27).

In our study, the incidence of these IMD putatively correlated with vaccines administration is low, but we should consider that our study has some limitations as well.

The study has been not designed to estimate the incidence and prevalence of IMD onset after COVID-19 or vaccination, but it is a real-life observational study conducted in a single tertiary referral center during a pandemic. Thus, the estimates could be affected by selection bias and the retrospective data collection and analysis.

In a large epidemiological study conducted in 5493 patients with RA, the propensity-scored weighted Poisson regression showed no significant association between arthritis flare and COVID-19 vaccination (28).

Conversely, in two different web-based surveys evaluating systemic rheumatic disease flare incidence post-SARS-CoV-2 vaccine, one study showed about 11.5% of patients and the other n. 66 patients who reported a worsening of the underlying disease closely correlated with the first and/or second dose of different vaccines (10, 29).

The most important finding of the latter case series, like in other reports (11–13), is that all the new IMDs or their exacerbations were mostly mild-to-moderate and, importantly, a short course of corticosteroids was sufficient to control disease manifestations in most cases.

Moreover, although the clinical manifestations were similar to those of the canonical IMDs, we found that conventional or disease-defining serological markers were usually absent.

These considerations open two important questions: how should these vaccine-induced IMDs be managed and for how long? What is their natural history?

These findings in vaccinated subjects potentially support the hypothesis that anti-spike antibodies elicited by vaccination and, likewise, those elicited by COVID-19 (15–19), may participate in triggering downstream signaling pathways common to those encountered in several immune-mediated disorders (13, 30, 31).

Considering the massive campaign of vaccination against SARS-CoV-2, the incidence of vaccine-associated IMDs seems however quite low (https://vaccine-safety-training.org).

In conclusion, this study shows that immunity against SARS-CoV-2 has the potential to stimulate heterogeneous immune-mediated reactions without specific serological autoimmunity. Our findings support the need to investigate on the role that anti-Spike antibodies may play in such cases and, most importantly, reinforce the current understanding that COVID-19 clinical manifestations are often sustained by the host immune system but not by SARS-CoV-2 itself (17, 18). The true incidence of IMDs, both as flares and as new onset manifestations, remains to be established. Larger epidemiological studies should be promoted globally to monitor these potential post-vaccination IMDs and to evaluate their real incidence and clinical significance.

## Data Availability Statement

The raw data supporting the conclusions of this article will be made available by the authors, upon reasonable request.

## Ethics Statement

Ethical review and approval was not required for the study on human participants in accordance with the local legislation and institutional requirements. The patients/participants provided their written informed consent to participate in this study.

## Author Contributions

ML, VP, VM, MG and DB contributed to conception and design of the study and to the clinical examination of the patients. RS, CM, LM organized the database and contributed to the clinical examination of the patients. DB performed the statistical analysis. GM, VP, and DB wrote the first draft of the manuscript. ML, GP, AO, and GM wrote sections of the manuscript. All authors contributed to manuscript revision, read, and approved the submitted version.

## Conflict of Interest

The authors declare that the research was conducted in the absence of any commercial or financial relationships that could be construed as a potential conflict of interest.

## Publisher’s Note

All claims expressed in this article are solely those of the authors and do not necessarily represent those of their affiliated organizations, or those of the publisher, the editors and the reviewers. Any product that may be evaluated in this article, or claim that may be made by its manufacturer, is not guaranteed or endorsed by the publisher.
